# Proteus Syndrome, a rare case with an unusual presentation: Case report

**DOI:** 10.1016/j.ijscr.2020.06.052

**Published:** 2020-06-13

**Authors:** N. Amer, J. Al Helal, M. Al Hajji, A. Al Abduljabbar, M. Al Arfaj, H. Al Sadery, A. Awadallah

**Affiliations:** King Fahad Hospital of the University, Imam Abdulrahman Bin Faisal University, Saudi Arabia

**Keywords:** Proteus Syndrome, Macrodactyly, Intestinal lipomatoses, Small bowel volvulus

## Abstract

**Introduction:**

Proteus Syndrome is a very rare syndrome, where only 200 cases has been reported so far.

**Presentation of case:**

We were presented with a 45 year male with features of chronic Intestinal Obstruction. On Examination he had a giant right middle finger (Fig. 1), giant left forearm, with amputated left hand, and massive abdominal distension with tenderness in upper abdomen.

**Discussion:**

Proteus Syndrome (PS) is a very rare syndrome, diagnosis of which is based on Biescker’s criteria, either one finding from Criteria A, or two from B, or three from C. Confirmation of the disease is by finding genetic variation in the AKT1 gene. In addition to the presence to some of the criteria in our patient, he also had numerous intestinal lipomatosis, involving almost most of his small intestine. This in addition to high riding caecum and a narrow long mesentery which contributed to the development of small Bowel volvulus.

**Conclusion:**

PS should be suspected whenever we encounter patients with macrodactyly and this should alert us to the possibility of other congenital anomalies.

## Highlights

• Proteus Syndrome is an extremely rare case where only 200 cases has been reported.
• Macrodactyly is a striking feature.
• Biesker’s criteria is a diagnostic tool, which assist in reaching the diagnosis.
• Confirmation is by finding genetic variation in AKT1 and/or PTEN gene.
• Our case had multiple intestinal lipomatosis which caused him small bowel volvulus.

## 1 Introduction

Proteus Syndrome (PS) is a very rare hamartomatous syndrome first described by the German paediatrician Rudolf Wiedemann 1983, and was named after the Greek sea God Proteus who could change his shape to evade capture [1].

We encountered this very rare syndrome who had this gigantic right middle finger and left arm who presented to us with small bowel volvulus. The pathology of the specimen showed that the whole length of the bowel was full of intraluminal lipomas. We decided to report this case because of its rarity, and around 200 cases only has been reported so far. There has been reports of intra-abdominal lipomas [2], but we could not find any report associating PS with intestinal lipomatosis.

There are certain clinical criteria for diagnosing PS in addition to chromosomal studies. In our case though we did not have chromosomal studies, but the clinical features suggest PS rather than any of the other differential diagnosis. The syndrome itself is not inherited and does not pass to the offspring, however, life expectancy is shorter than expected specially if it is not the mild form.

This paper has been reported in line with the SCARE criteria [3].

## 2 Case report

**Fig. 1:**
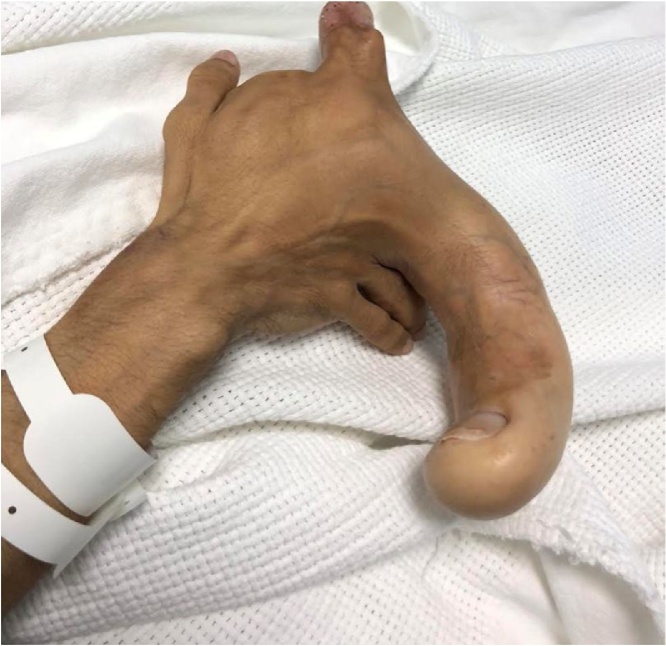
Right giant middle finger.

A 45 years old Saudi male was admitted because of symptoms of Intestinal obstruction started 6 days prior to presentation. He presented with constipation and vomiting. He also noticed that his abdomen was getting distended for the past four months. His past medical history was not significant. On examination, his vital signs were stable, his abdomen was very distended and tympanic, but not tender. Strikingly he had a gigantic right middle finger (Fig. 1). The left hand was amputated because of the massive size which was too heavy to lift. The whole left arm was hypertrophied and he also had a large diffuse lipoma occupying his left chest wall. His face was elongated and he had mild slanting eyes. The speech was very coherent and he maintained good mentation with Glasgow score of 15 all the time. There was no obvious abnormality in the lower limbs and no evidence of any dermatological changes. Though not married, he was fully independent, engaged in a full time job, running a business with 300 employees under his care, and he drove his own car. His blood investigations showed a white count of 13.2, Hb of 12.1 gm/dl, platelets of 252,000 per micro l. His PT, PTT and INR were within the normal range, however his sodium was 107 meq/dl, and his Cl was 75 meq/dl and his potassium was 3.6 meq/dl. Rest of his blood test showed normal liver function and normal renal function. Computer Tomographic scan of his abdomen confirmed diagnosis of small bowel obstruction. He was admitted for conservative treatment initially, however in the next 24 h he became unwell and his abdomen became more tender, more distended with guarding in the upper right quadrant. His plain XR of his abdomen confirmed small bowel obstruction and the patient was taken to the operating theatre where an exploratory laparotomy was done. The findings were hugely dilated small bowel forming a volvulus which was twisted 180 degrees around its elongated mesentery, the caecum and the appendix were found in left upper quadrant. The point of twist was just 8 cm distal to the ileocecal junction. There was also a small perforation right at twisting point.

**Fig. 2:**
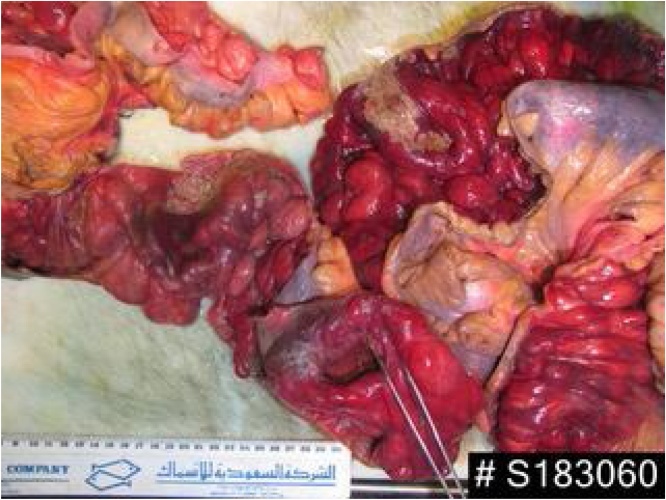
Part of the terminal ilieum showing numerous giant lipomatosis.

**Fig. 3:**
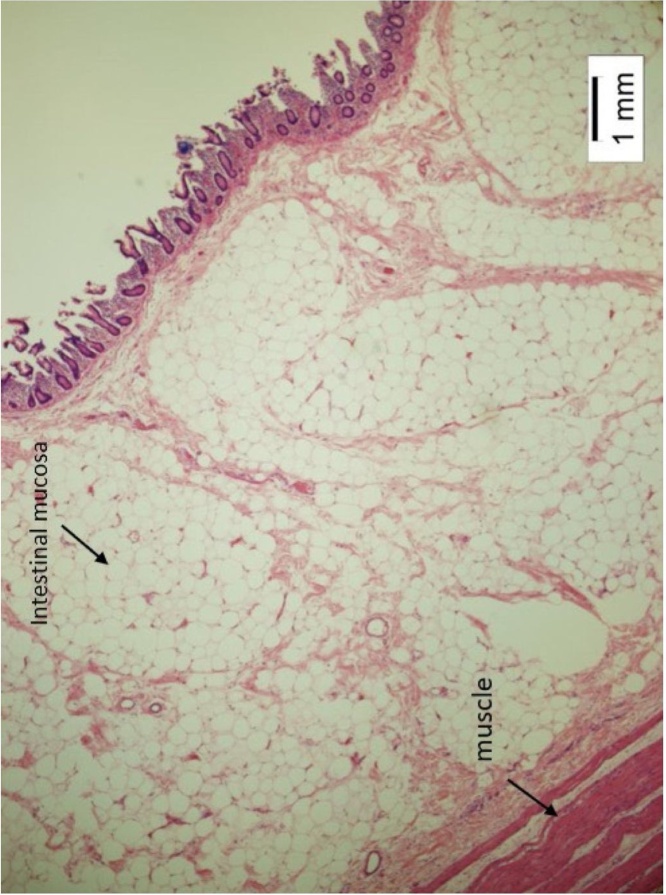
Showing fat cells in the cross section of one of the lipomas.

Resection of the twisted bowel was done due to its dusky colour and lack of motility and shine, and opening the specimen revealed numerous intraluminal lipomas (Figs. 2, 3). With end to end anastomosis was done, 170 cm were removed of ileum, and over 200 cm of small bowel was left intact. Patient was admitted in the Intensive care unit where he spent a stormy 40 days, during which time he was taken back to the operating theatre and a second look was done, where an anastomotic leak was discovered. The anastomosis was taken down and an ileostomy and a mucus fistula was created. This was reversed five months later, prior to his discharge mainly because of his severe weight loss from the continuous loss from the stoma, which could not be compensated by his naso-gastric feed, and partly because of the local complication of the stoma. During his hospital stay he developed few grand mal fits which were controlled using Keppran and valproic acid. His brain CT revealed pontine demylination, which most likely were due to a rapid correction of his hyponatraemia.

## 3 Discussion

Proteus Syndrome (PS) is extremely rare with an estimated around 200 cases only been reported, with an incidence of 1 in 1,000,000 [2,4]. It is a sporadic disorder that manifest as an asymmetric disproportionate overgrowth of any connective tissue such as bone, fat or epidermal nevi in a mosaic or patchy pattern. The syndrome was named after the Greek God Proteus, who could change his shape at will to evade capture [1,2]. We encountered this interesting gentleman with his giant right middle finger who presented to us with a picture of small bowel obstruction, which turned out to be due to small bowel volvulus, most likely caused by the heavy weight of the intestine which was full of intraluminal lipomas and the long narrow mesentery, in addition to his high riding caecum.

The following criteria’s are pre-requisite for the diagnosis of PS as described by Biesecker [4,5]:

Category A: cerebriform connective tissue nevus (CCTN)

Category B

• Linear epidermal nevus
• Asymmetric, disproportionate overgrowth of limbs, Hyperostosis of the skull, external auditory canal, Megaspondyldysplasia (abnormal growth of the vertebrae), viscera: spleen/thymus
• Tumors: ovarian cystadenoma, Parotid monomorphic adenoma.

Category C

• Dysregulated adipose tissue (either of the following): Lipomatous overgrowth, Regional lipoatrophy
• Vascular malformations (one of the following) : Capillary malformation, Venous malformation, lymphatic malformation, lung bullae
• Facial phenotype (all of the following): Dolichocephaly, long face, Down slanting palpebral fissures and/or minor ptosis, depressed nasal bridge, Wide or anteverted nares, Open mouth at rest

The diagnosis can be established if we have all of the General criteria (Mosaic distribution of lesions, Sporadic occurrence, progressive course) and specific criteria from categories A-C:

• One from A or
• Two from B or
• Three from C

We diagnosed our case as PS based on the Gigantism of his right middle finger (Fig. 1), asymmetry in the left arm and giant lipoma in his left chest wall in addition to his elongated face and slight slanting of his palpebral fissures. The other differential diagnosis include the following:

• Macrodystrphia lipomatosa [4,6]
• Fibrolipomatous hamartoma [5,7]
• Neurofibromatosis [6,7]
• Lymphangiomatosis [4,6]
• Klippel-Trenouny Weber [7]
• Maffucci disease [1,7]
• Ollier disease [7]
• Bannay Zonana Syndrome [5]
• Multiple lipmatosis syndrome [5]
• PTEN hamartoma tumour syndrome (PHTS) [5]
• CLOVES Syndrome (Congenital/Lipomatous asymmetric overgrowth of the Trunk, lymphatic, capillary, venous and combined type vascular malformation, epidermal nevi skeletal muscle and spinal anomalies) [5]
• Hemi-hyperplasia [5]

Unfortunately in our institution we do not have the facility of genetic analysis in order to confirm PS, which is usually associated with variation in AKT1 [[7], [8], [9]]. Mutation of PTEN has also been reported. The disorder, however, is not inherited, and does not run in the family. Classically patients with PS present with wide range of severity [5], they usually have little or No manifestation at birth. Asymmetric growth of hands and feet start from age of six to eighteen months and full over growth of the area concerned occur at the age of six. The presence of cerebriform connective tissue nevi is pathognomonic. These are firm skin lesions with brain like sulci and gyri (hence the term cerebriform). Many PS patients develop regional lipomatous overgrowth and lipo atrophy, this was noticed in the left side of our patient’s chest. Many individuals develop cutaneous capillary malformation and prominent varicosities, large and complex vascular malformation. PS patient are at risk of developing deep venous thrombosis and pulmonary embolism [5], benign tumours including meningioma, ovarian cystadenoma, parotid monomorphic adenoma and rarely may develop a bullous pulmonary disease.

The management is complex, and may involve epiphysiolysis, osteotomy, surgery for scoliosis, DVT prophylaxis, dermatological care of the CCTN, surgical resection of overgrowth of lipomatous tissue, screening for tumours and psychosocial counselling. DNA Banking has been advised (typically extracted from white cell) for possible future use.

## 4 Conclusion

PS is a very rare syndrome and once you come across a patient with giant fingers or hands, we should suspect, and look for other anomalies that may be of more serious consequences. Our patient was very lucky, despite passing through very stormy four laparotomies, and 40 days in the intensive care, and total of four months in the hospital, he was discharged home in good condition.

Written informed consent was obtained from the patient for publication of this case report and accompanying images.

## Declaration of Competing Interest

No conflict of interest.

## Sources of funding

No funding required.

## Ethical approval

This is a case report, No ethical approval required.

## Consent

Written informed consent was obtained from the patient for publication of this case report and accompanying images. A copy of the written consent is available for review by the Editor-in-Chief of this journal on request.

## Author contribution

**Dr Nasser Amer**: Main author, treating physician, main writer, helped with the analysis and collection of all data.

**Dr Jumana Waleed Al Helal**: collected the data from the patient notes and all the figures.

**Dr Abdulrahman Abduljabaar**: Collected and analysed the literature.

**Dr Mohammed Al Haji**: Collected and summarised the literature.

**Dr Mosaab Al Arfaj**: Collected and analysed and summarised the literature, and helped with the collection of the notes.

**Dr Humood Alsadery**: Helped with management and the writing of the paper.

**Awadia Abdallah**: helped with the preparation of the pathology slides and reporting it and the literature concerning the case.

## Registration of research studies

NA.

## Guarantor

Dr Tarig M Abd Al Hafiz.

Assistant Professor General Surgery, Consultant General Surgery, King Fahad Hospital of the University, Al Khobar, KSA.

taralhafiz@gmail.com
